# SCD5 expression correlates with prognosis and response to neoadjuvant chemotherapy in breast cancer

**DOI:** 10.1038/s41598-021-88258-9

**Published:** 2021-04-26

**Authors:** Weipeng Zhao, Linlin Sun, Xichuan Li, Jun Wang, Ye Zhu, Yan Jia, Zhongsheng Tong

**Affiliations:** 1 Department of Breast Cancer, Tianjin Medical University Cancer Institute and Hospital, National Clinical Research Center for Cancer, Key Laboratory of Cancer Prevention and Therapy, Tianjin, People’s Republic of China; 2 Tianjin Key Laboratory of Animal and Plant Resistance, College of Life Sciences, Tianjin Normal University, Tianjin, 300387 People’s Republic of China; 3 Department of Oncology, The First Affiliated Hospital of Shandong First Medical University, Jinan, People’s Republic of China

**Keywords:** Cancer, Biomarkers, Oncology

## Abstract

Neoadjuvant chemotherapy (NACT) represents a standard option for breast cancer. Unfortunately, about 55–80% of breast cancer patients do not have a favorable response to chemotherapy. Highly specific tumor biomarker that can predict the pathological response to neoadjuvant chemotherapy is lacking. Stearoyl-CoA desaturase 5 (SCD5) is an integral membrane protein of the endoplasmic reticulum that participates in lipid metabolism. Previous studies on the role of SCD5 in human cancers drew different conclusions. Therefore, the role of SCD5 in breast cancer remains unclear. Our study aims to understand its expression signature, prognosis value and correlation with pathological response to NACT in breast cancer using bioinformatics from public databases. Analysis of samples from public databases showed that SCD5 expression was down-regulated in some human cancers including breast cancer, and low expression of SCD5 was associated with more aggressive breast cancer phenotypes. Survival analysis revealed that SCD5 expression was related to prognosis in breast cancer. Integrated analysis of multiple public datasets indicated that SCD5 expression signature was associated with pathological response to NACT, particularly in TNBC. Based on functional enrichment analysis, the most affected biological functions in high SCD5-expressing breast cancer tissues were involved in negative regulation of cell cycle. Moreover, a significantly negative correlation between SCD5 expression and several cell cycle regulators was noted. Taken together, SCD5 was involved in the development and progression of breast cancer and might be a predictive biomarker for response to NACT. In conclusion, SCD5 could serve as a predictive biomarker of pathological response to NACT and play a carcinostatic role in breast cancer. These results provided us with clues to better understand SCD5 from the perspective of bioinformatics and highlighted the clinical importance of SCD5 in breast cancer, especially triple negative breast cancer (TNBC).

## Introduction

According to the latest estimates of cancer incidence and mortality produced by the International Agency for Research on Cancer, breast cancer has surpassed lung cancer as the most commonly diagnosed cancer, and remained the leading cause of cancer death among females^1^. Breast cancer that lacks the expression of estrogen receptor (ER), progesterone receptor (PR), and human epidermal growth factor receptor 2 (HER2) is classified as TNBC. Compared to other breast cancers, TNBC accounts for 15% of all breast cancers and is associated with stronger invasiveness, higher risk of early recurrence rate and inferior survival^2^. NACT, as a preoperative treatment to downgrade the tumors, was first used for locally advanced and inoperable breast cancer and now it has been widely used in operable breast cancer, especially in TNBC. Pathological complete response (pCR) is defined as the absence of residual invasive cancer on pathological evaluation of breast primary and lymphonodes after neoadjuvant chemotherapy^3^. Several studies have revealed that pCR could serve as a predictor of long-term survival^4–6^. The prognostic value of pathologic response makes NACT become the current standard option for patients with breast cancer. However, not all of patients with breast cancer have a favorable pathological response to NACT^7^. It is well recognized that some intrinsic molecular and clinicopathological characteristics of tumor biology can affect the response degree to neoadjuvant chemotherapy. However, the highly specific biomarkers that can predict the pathological response to NACT and the prognosis of breast cancer have not been clearly defined.

Increased lipogenesis is one of the most important metabolic markers of cancer cell. The proliferation of cancer cells requires the continuous formation of new cell membranes, and the formation of membrane lipids depends on the synthesis of fatty acids^8^. The fatty acids composition of cell lipids is mainly regulated by stearoyl-CoA desaturase (SCDs). SCDs, as the endoplasmic reticulum-resident integral membrane proteins, catalyze the formation of monounsaturated fatty acids from saturated fatty acids. Four SCD isoforms (SCD1–SCD4) have been identified in mice and two SCD isoforms (SCD1 and SCD5) in human^9^. SCD1 is confirmed to be up-regulated in the majority of cancers and participates in cell cycle and tumor cell migration^10,11^. Though displaying similar desaturation activities, the biological role of SCD5 in human cancers remains unclear. Previous studies found that SCD5 could protect against malignancy and reverse the epithelial-mesenchymal-like process in melanoma^12,13^. While, SCD5 was also reported to be up-regulated in uveal melanoma, and high expression of SCD5 was related to poor prognosis^14^. In addition, Chen et al. found that the SCD5 expression was down-regulated in patients who achieved pCR after neoadjuvant chemotherapy compared to patients who did not achieve pCR^15^. Here, this study was performed to comprehensively analyze SCD5 expression characteristics, prognostic value and correlation with pathological response to NACT for better understanding the clinical significance of SCD5 in breast cancer.

## Materials and methods

### NCBI gene expression omnibus (GEO)

GEO (http://www.ncbi.nlm.nih.gov/geo/) is a worldwide resource for gene expression studies. It is used to archive and distribute high-throughput gene expression and other functional genomics datasets^16^. GEO2R (https://www.ncbi.nlm.nih.gov/geo/geo2r/) and the software R (The R Programming Language, version 4.0.4) were performed to process the downloaded data. Then, the data was calibrated and standardized by software R.

### The Cancer Genome Atlas (TCGA)

The Cancer Genome Atlas (TCGA) is a publicly available database, which has molecularly characterized over 20,000 primary cancer and matched normal samples spanning 33 cancer types. TCGA provided genomic, epigenomic, transcriptomic, and proteomic data^17^.

### Genotype-tissue expression (GTEx)

GTEx is a web-based, freely accessible database, which provided gene expression data from 53 normal tissue sites across nearly 1000 people by RNA sequencing. RNA-seq data including GTEx and TCGA data were derived from UCSC Xena data hubs (http://xena.ucsc.edu/) for a pan-cancer differential expression of SCD1 and SCD5.

The Human Protein Atlas (HPA) HPA (https://www.proteinatlas.org/), aims to map human proteins in cells, tissues and organs, which is available for studying protein localization and expression in human tissues and cells^18^.

### Gene expression profiling interactive analysis (GEPIA)

GEPIA (http://gepia.cancer-pku.cn/) is a web server for analyzing the RNA sequencing expression data of 9736 tumors and 8587 normal samples from the TCGA and the GTEx data, it provides customizable functions such as tumor/normal differential expression analysis, profiling according to cancer types or pathological stages, patient survival analysis, similar gene detection, correlation analysis and dimensionality reduction analysis.

### Oncomine

The Oncomine platform (https://www.oncoline.org/; version 4.5), a cancer microarray database and web-based data-mining platform, aims at facilitating discovery from genome-wide expression analyses and compare the transcriptome data in most major types of cancer with normal counterparts^19^. To date, Oncomine contains gene expression profile data of 86,733 samples from 715 datasets.

### Kaplan–Meier plotter

The Kaplan–Meier-plotter online software (http://kmplot.com/analysis/) is an online database including gene expression data and clinical data. The prognostic value of the mRNA expression of SCD1 and SCD5 in breast cancer was evaluated using the Kaplan–Meier plotter^20^.

### Functional enrichment and gene ontology analysis

The significant pathways influenced by SCD5 were analyzed by using KEGG pathway database (https://www.kegg.jp)^21–23^. Software R (version 4.0.4) was used to find SCD5-related DEGs. The functional enrichment and GO annotations of overlapped DEGs were analyzed by Metascape database (http://metascape.org).

### Statistical analysis

The Wilcoxon rank-sum and t tests were used to analyze the association between gene expression and clinicopathological characteristics. Pearson correlation was performed to analysis the correlation between SCDs and HER2/ERBB2 expression. R version 4.0.4 and GraphPad Prism (version 8.0.1) were applied for statistical analysis and image production. *P* value < 0.05 was determined to be significantly different (two-sided).

### Ethical approval

All data in this manuscript was collected under the guidelines approved by Tianjin Medical University Cancer Institute and Hospital’s institutional review board and complying with the current laws in China.

## Results

### SCD5 expression in breast cancer

**Figure 1:**
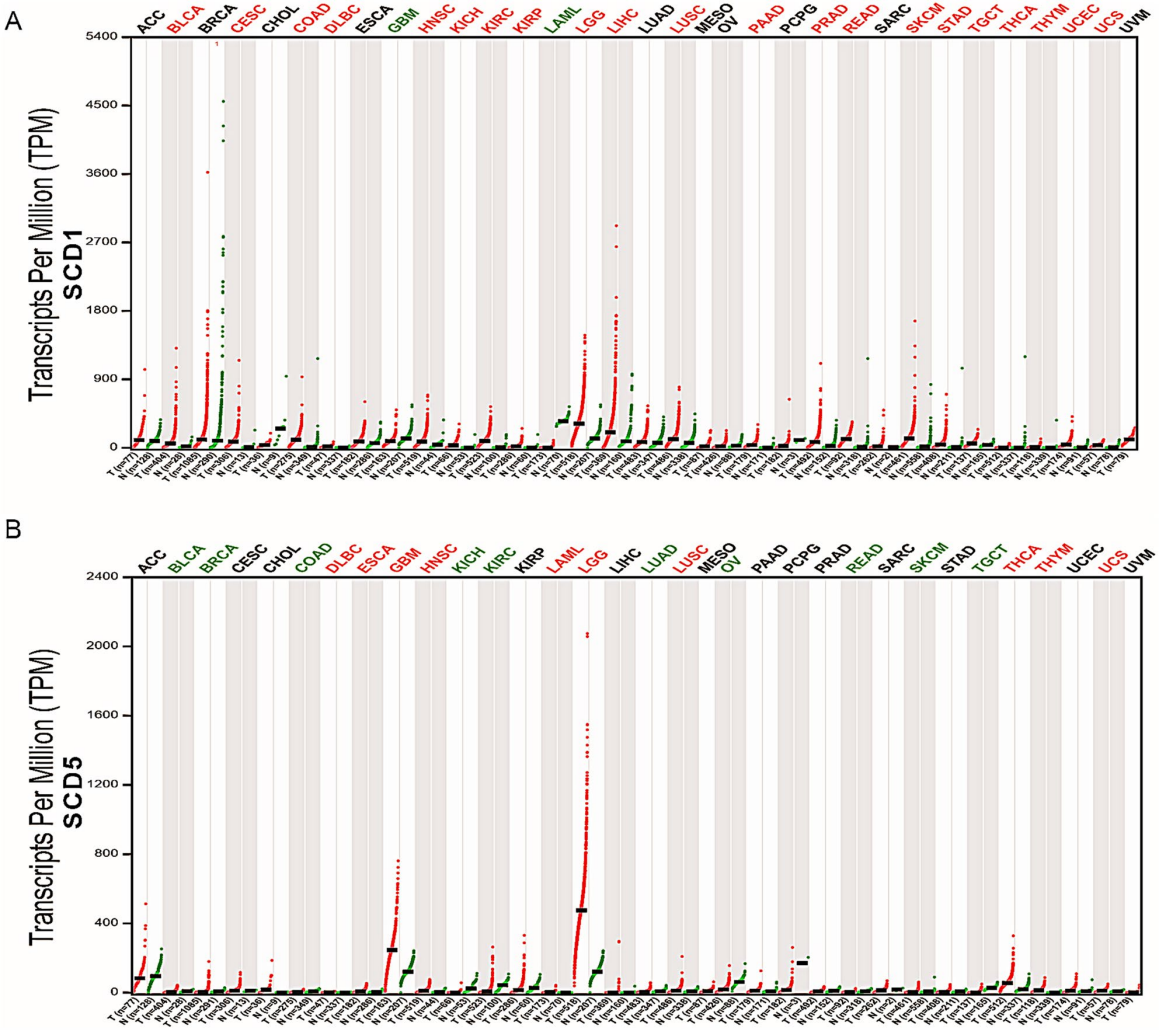
The mRNA expression of SCD1 and SCD5 in human cancers. (**A**) The mRNA expression of SCD1 between tumor and normal tissue was assessed using tissues from TCGA and GTEx. (**B**) The mRNA expression of SCD5 between tumor and normal tissue was assessed using tissues from TCGA and GTEx. Cancer types in red font indicate that SCD1 or 5 mRNA expression was upregulated in tumors compared to their normal counterparts. Cancer types in green font indicate that SCD1 or 5 mRNA expression was down-regulated in cancers compared to their normal counterparts. *T* tumor, *N* normal.

**Figure 2:**
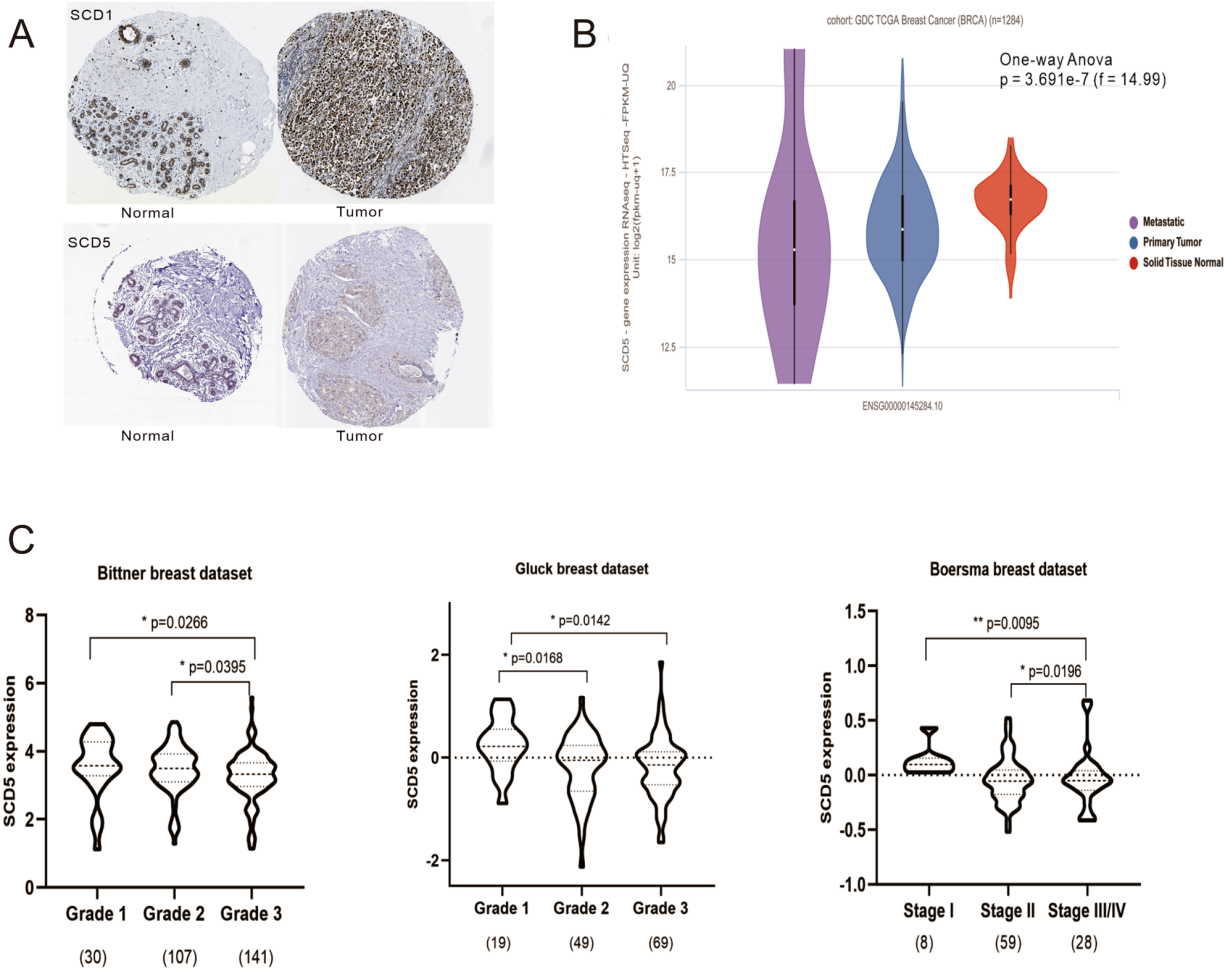
SCD5 expression in breast cancer. (**A**) Representative result of SCD1 and 5 expression using samples from the Human Protein Atlas. (**B**) SCD5 expression in breast cancer and normal breast tissue using samples from TCGA. (**C**) SCD5 expression in different histological grades and pathological stages of breast cancer using datasets from Oncomine (*p value ≤ 0.05; **p value ≤ 0.01; ***p value ≤ 0.001).

**Figure 3:**
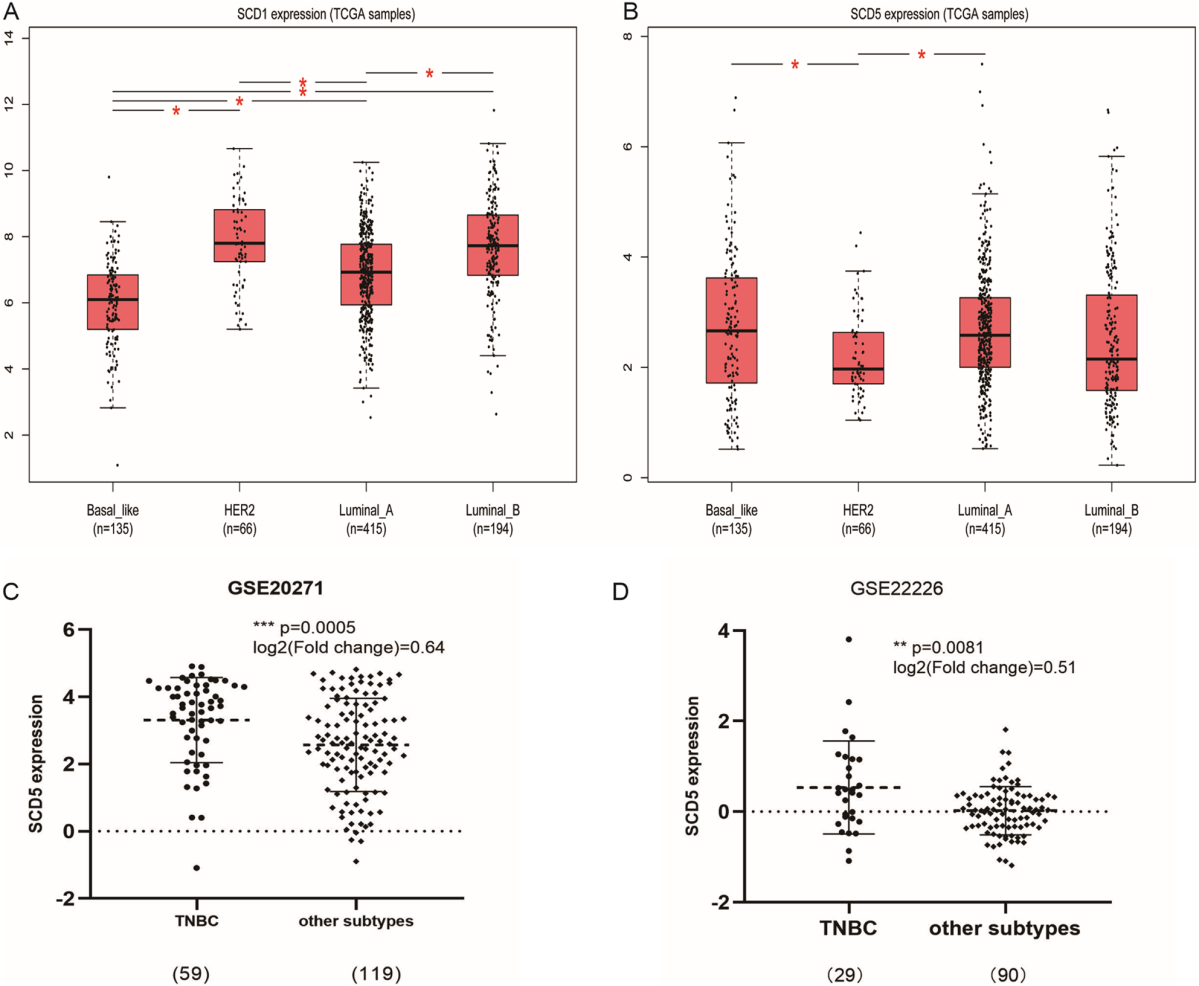
The correlation between SCDs (SCD1 and SCD5) mRNA expression and breast cancer molecular subtypes. (**A**, **B**) SCD1 and SCD5 mRNA expression in different molecular subtypes of breast cancer by using TCGA samples. (**C**, **D**) SCD5 mRNA expression in different molecular subtypes of breast cancer by using GEO data.

Normal tissue samples from GTEx were performed to investigate SCD5 mRNA expression among different human organs (Figure S1). The results showed that SCD5 mRNA expression was enriched in brain and adrenal gland. In breast tissue, the mRNA of SCD5 was moderately expressed. We then used integrated samples from TCGA and GTEx to identify SCD5 mRNA expression in human cancers, and compared expression differences of SCD5 and SCD1 among different human cancers. Collectively, the analysis showed that SCD5 had different mRNA expression profiles in different cancer types (Fig. 1B). SCD5 was downregulated in breast invasive carcinoma (BRCA), bladder urothelial carcinoma (BLCA), colon adenocarcinoma (COAD), kidney chromophobe (KICH), kidney renal clear cell carcinoma (KIRC), lung adenocarcinoma (LUAD), ovarian serous cystadenocarcinoma (OA), rectum adenocarcinoma (READ), skin cutaneous melanoma (SKCM), and testicular germ cell tumors (TGCT) but was upregulated in lymphoid neoplasm diffuse large B-cell lymphoma (DLBC), brain lower grade glioma (LGG), esophageal carcinoma (ESCA), glioblastoma multiforme (GBM), head and neck squamous cell carcinoma (HNSC), thyroid carcinoma (THCA), uterine carcinosarcoma (UCS), thymoma (THYM), lung squamous cell carcinoma (LUSC) and acute myeloid leukemia (LAML). Different from SCD5, SCD1 mRNA expression was upregulated across most human cancer types (Fig. 1A). Though the two isoforms displayed similar desaturation activities but have different distributions in human cancers. In some cancer types, such as DLBC, HNSC, LUSC, UCS, THCA, THYM and LGG, both SCD1 and SCD5 mRNA expression were upregulated. While, different mRNA expression profiles of SCD1 and SCD5 were observed in the majority of cancer types. For breast cancer, SCD5 mRNA expression was significantly downregulated in tumors compared with normal tissues, but there was no significant difference in SCD1 mRNA expression between breast tumors and normal tissue. In order to explore the roles of SCD5 in breast cancer, multiple analyses were performed on the Human Protein Atlas, TCGA and GEO databases. The result of immunohistochemistry revealed that highly expressed SCD5 was found in breast normal tissues compared to tumor tissues, which was opposite to SCD1 (Fig. 2A). This significant difference was confirmed by the expression analysis of SCD5 in primary breast cancer samples and normal breast samples derived from TCGA data (n = 1284) (*p* = 3.691e−7) (Fig. 2B). In addition, we also analyzed the mRNA expression of SCD5 in breast cancer patients with different molecular and clinicopathological characteristics. The results revealed that SCD5 mRNA expression was under-expressed in high histological grade and late-stage breast cancer (Fig. 2C). Unlike SCD5, we did not observe significant differential SCD1 mRNA expression among different breast cancer histological grades or pathological stages (not shown in this paper). However, we found that the ratio SCD5/SCD1 was significantly different in different histological grades and pathological stages of breast cancer (Figure S2). The analysis showed that the ratio SCD5/SCD1 was significantly upregulated in low histological grade and early-stage breast cancer compared to high histological grade and late-stage breast cancer. According to the expression status of ER, PR and HER2, the primary breast tumor was divided into different subtypes with different outcomes and response to treatment^24^. Tumor samples from TCGA and GEO databases were used to explore the distribution of SCD5 mRNA expression in different breast cancer molecular subtypes. The analysis of TCGA samples showed that SCD5 mRNA expression was significantly higher in TNBC and Luminal A subtypes compared to other molecular subtypes (*p* < 0.05) (Fig. 3B). However, the analysis found that SCD1 mRNA expression was significantly upregulated in HER2 enriched breast cancer (*p* < 0.05) (Fig. 3B). In GEO data, upregulated SCD5 mRNA expression was observed in TNBC compared to other breast cancer subtypes (log2[Fold change] > 0.5, *p* < 0.05) (Fig. 3C, D). Taken together, these findings indicated that down-regulated SCD5 expression was related to more aggressive breast cancer phenotypes, such as high histological grade, late stage and HER2 overexpression.

### Correlation analysis between mRNA expression of SCD5 and prognostic value

**Figure 4:**
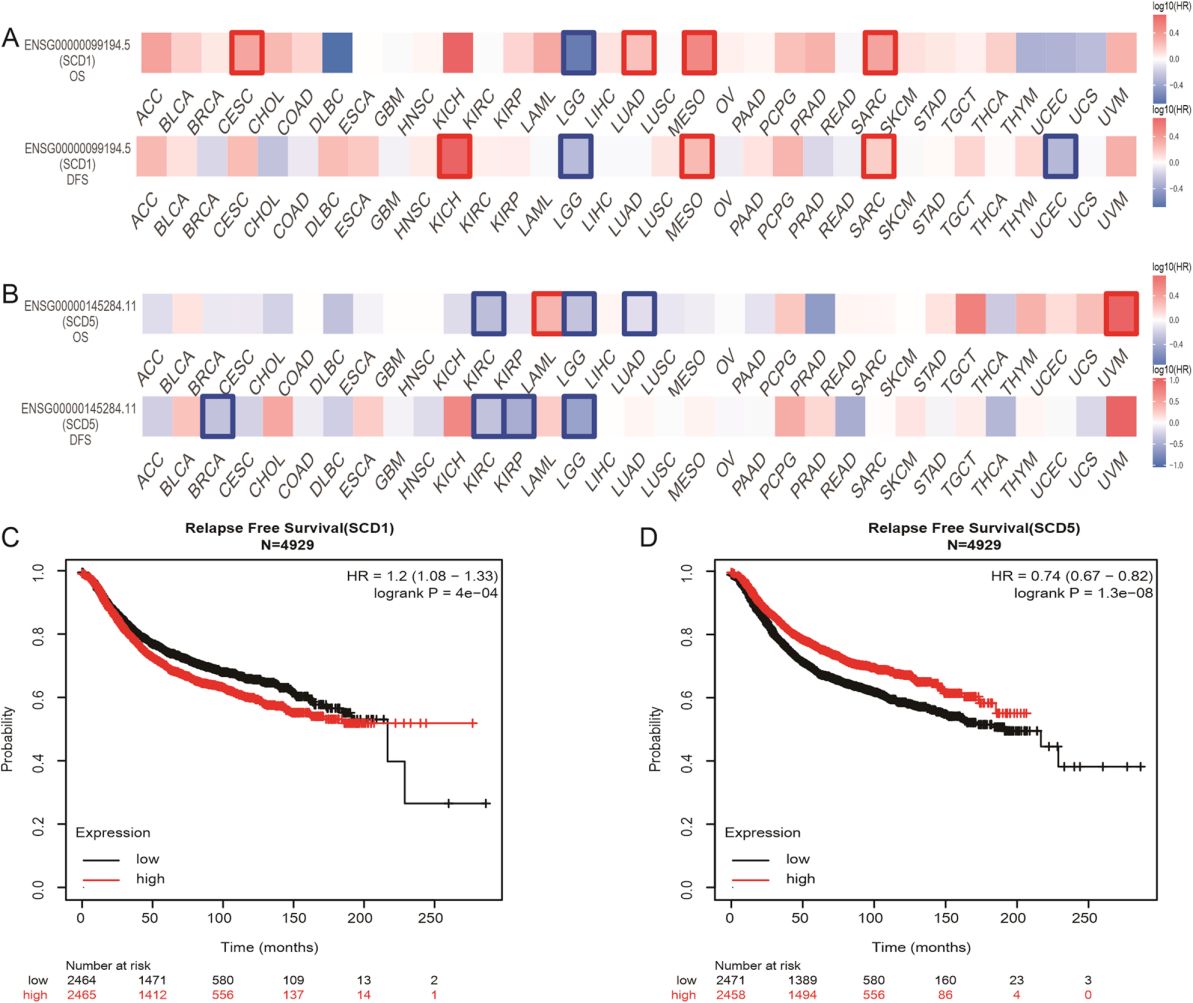
The prognostic significance of SCD1 and SCD5 in human cancers. (**A**, **B**) Survival map of SCD1 and SCD5 in human cancers by using samples from TCGA. (**C**, **D**) Survival analysis of SCD1 and SCD5 expression in breast cancer by using Kaplan Meier plotter.

We further explored the correlation between the gene expression and survival in breast cancer to better understand the clinical significance of SCD5. Samples from TCGA were performed to carry out the survival map of SCD1 and SCD5 in human cancers. The results showed that SCD5 had different prognostic values in different cancer types. Upregulated SCD5 expression was associated with longer overall survival (OS) in KIRC, LGG, and LUAD but was associated with shorter OS in LAML and UVM. For breast cancer, the results showed that there was no correlation between SCD5 expression and OS. While, upregulated SCD5 expression was related to longer disease free survival (DFS) in BRCA. Unlike SCD5, there was no significant correlation between SCD1 expression and OS/DFS in breast cancer (Fig. 4A, B). We also investigated the effect of SCD1 and SCD5 expression on prognosis in breast cancer by using Kaplan–Meier plotter dataset (Fig. 4C, D). Upregulated SCD5 expression was found to be associated with longer relapse free survival (RFS) in breast cancer (HR = 0.74, 95% CI 0.67–0.82, *p* = 1.3e−08). While, upregulated SCD1 expression was found to be associated with shorter RFS in breast cancer (HR = 1.2, 95% CI 1.08–1.33, *p* = 4e−04). Neither SCD1 nor SCD5 was found to be significantly related to OS in breast cancer by using Kaplan–Meier plotter dataset (not shown in this paper).

### Relationship between mRNA expression of SCD5 and pathological response to neoadjuvant chemotherapy

**Figure 5:**
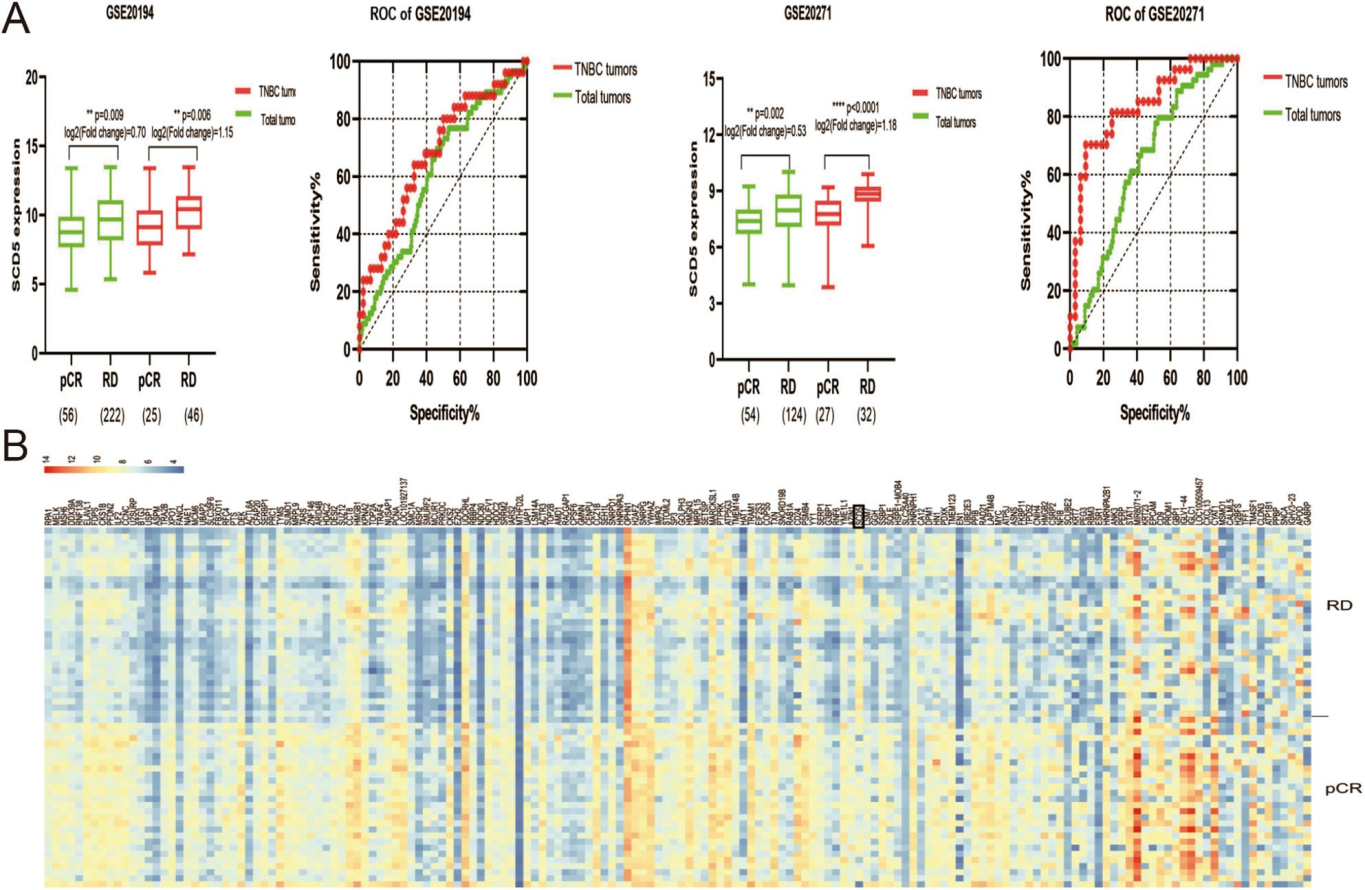
Relationship between SCD5 expression and response to neoadjuvant chemotherapy. (**A**) SCD5 expression in pCR cohort (patients with pathological complete response to neoadjuvant chemotherapy) and RD cohort (patients with residual disease after neoadjuvant chemotherapy) using datasets from GEO. ROC curves for SCD5 expression levels to discriminate the breast cancer patients (especially TNBC patients) who achieve pCR from non-pCR after neoadjuvant chemotherapy using data from GEO datasets. (**B**) The DEGs of GSE25055 dataset (TNBC samples) were shown as cluster heatmaps. The DEGs were generated from pCR-group and RD-group. The red points represented upregulated genes (logFC > 1), while the blue points represented down-regulated genes (|log fold change (FC)|> 1, *p* < 0.05).
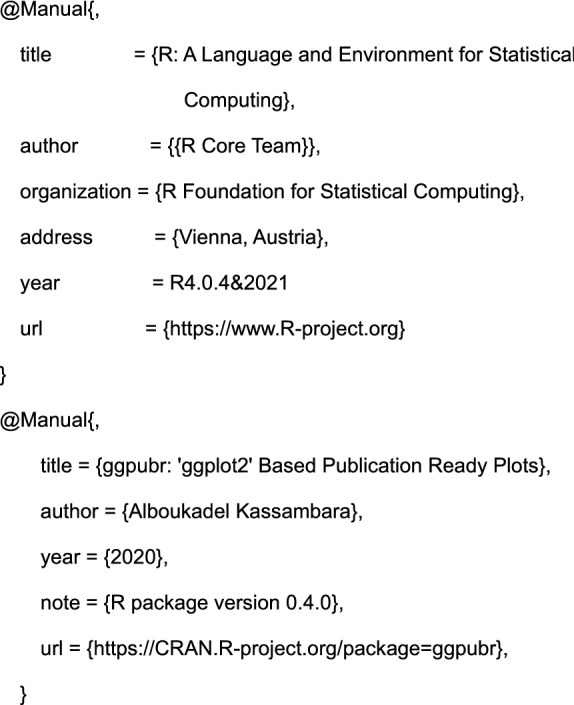

HER2/ERBB2 (erb-b2 receptor tyrosine kinase 2), a member of the epidermal growth factor receptor family, is amplified/overexpressed in 15–20% of breast cancers and associated with poor prognosis^25^. Some correlative studies of breast cancer indicated that the overexpression/amplification of HER2/ERBB2 was associated with a significant benefit from paclitaxel/doxorubicin^26–29^. Thus, compared to patients with HER2-negative breast tumors, patients with HER2-postive breast tumors might get more favorable response from NACT. Previous study showed that the expression of SCD5 varied between pCR cohort and non-pCR cohort^15^. In this article, we have already found that the SCD5 expression in triple-negative breast cancer was higher than that in other breast cancer subtypes using public datasets. Then, we further explored the relationship between SCD5 expression and HER2/ERBB2 expression by using Oncomine (Gluck breast cancer) and GEO (GSE20194) datasets. As shown in Figure S3, SCD5 expression was negatively correlated with HER2/ERBB2 expression (*p* < 0.05). By contrast, SCD1 expression was positively correlated with HER2/ERBB2 expression (*p* < 0.05). Thus, we speculated that high SCD5 expression characteristic was associated with unfavorable response to chemotherapy in breast cancer. We then performed microarray datasets downloaded from GEO (GSE20194, GSE20271 and GSE25055) to further define the value of SCD5 in curative effect assessment of anthracycline/taxane-based NACT for breast cancer. The results showed that SCD5 was differentially expressed between responders and non-responders (Fig. 5A, *p* < 0.05). The SCD5 exhibited a significant down-regulated expression in responders in comparison to non-responders. We also used TNBC samples from GSE25055 datasets to analyze the pathological response-related differentially expressed genes. The heat map showed that SCD5 expression was significantly upregulated in RD (residual disease) group compared with pCR group (Fig. 5B). Besides, In TNBC, the fold change of differential SCD5 expression between pCR group and RD group was higher than that in total tumors. Furthermore, receiver operating characteristic curve (ROC curve) analysis indicated that SCD5 could be a good predictor of pCR in patients with breast cancer, especially in TNBC (Fig. 5A, Table 1, *p* < 0.05). However, there was no significant correlation between SCD1 expression and chemotherapy response (Figure S4).

**Table 1:** ROC analysis of SCD5 in pretreatment tumor biopsy samples for predicting pCR.

GEO datasets	No. of patients			AUC	*p* value	95% CI
	Total	No. of pCR	No. of non-pCR			
GSE20194(Total tumors)	278	56	222	0.6123	0.0094	0.5317–0.6929
GSE20194(TNBC tumors)	71	25	46	0.6817	0.0119	0.5496–0.8139
GSE20271(Total tumors)	178	54	124	0.6441	0.0023	0.5612–0.7271
GSE20271(TNBC tumors)	59	27	32	0.8368	< 0.0001	0.7329–0.9407

### Potential pathways influenced by SCD5

As mentioned earlier, downregulated SCD5 expression was related to more aggressive breast cancer phenotypes, such as high histological grade, late stage and HER2 overexpression. Besides, we also found that the compared with wild type, SCD5 expression was down-regulated in several oncogene/tumor suppression genes mutation type (Figure S5) such as KRAS (Kirsten Rat Sarcoma Viral Oncogene Homolog), BRAF (B-Raf proto-oncogene, serine/threonine kinase and CDKN2A (cyclin dependent kinase inhibitor 2A). Therefore, we speculated that SCD5 might play a role in inhibiting the development and progression of tumor cells. KEGG pathway database and Metascape database were used to further explore the potential SCD5 regulatory mechanisms. Analysis of KEGG pathway showed that besides fatty acid metabolic pathway, SCD5 was also involved in peroxisome proliferator-activated receptor (PPAR) and AMP-activated protein kinase (AMPK) signaling pathways (Table 2), which were related to lipid metabolism as well as the inhibition of the multiplication of tumor cell^30–33^. In addition, AMPK was also closely involved in cancer drug resistance^34^. Though SCD1 was involved in similar signaling pathways (not shown in this paper), we did not observe any other clinical implications of SCD1 in breast cancer expect for prognostic value by using public databases.

**Table 2:** KEGG pathway of SCD5.

Pathway	Description	*p* value
hsa01040	Biosynthesis of unsaturated fatty acids	0.003
hsa01212	Fatty acid metabolism	0.007
hsa03320	PPAR signaling pathway	0.009
hsa04152	AMPK signaling pathway	0.01

**Figure 6:**
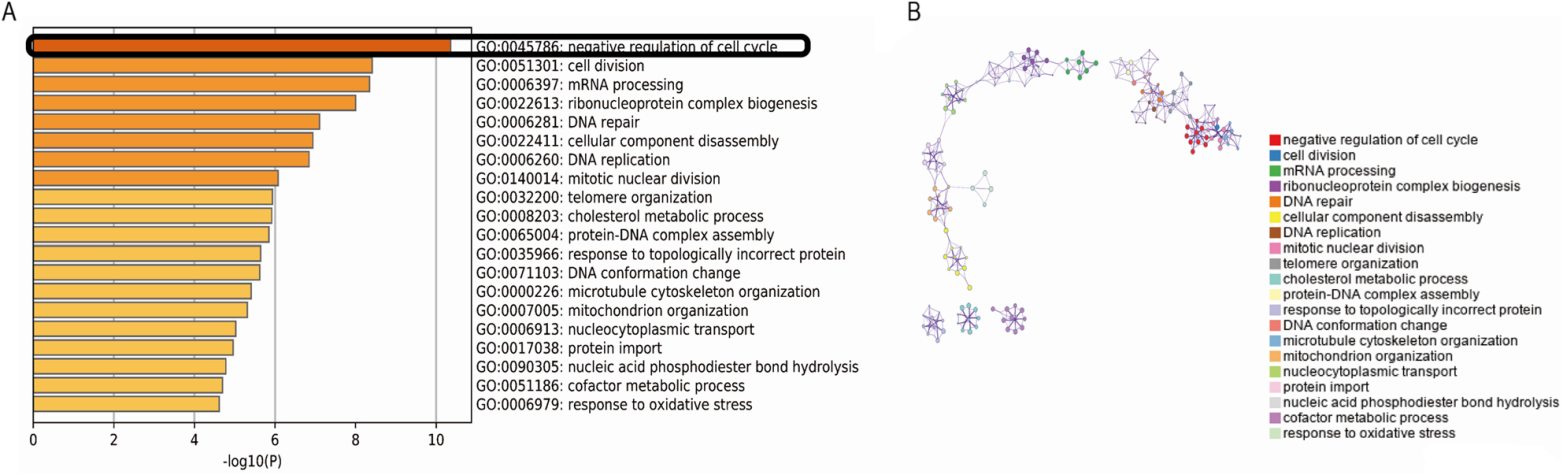
GO annotation and functional enrichment of SCD5-related differential expressed genes (DEGs). (**A**) GO biological process analysis of SCD5-related DEGs was performed by using Metascape with the criteria of *p* value < 0.01. (**B**) Network plot of the relationships among GO terms. Nodes represented enriched terms colored by its cluster identifier.

To further study the possible molecular functions and biological networks of SCD5, samples from GEO datasets (GSE25055 and GSE25065) were divided into two groups according to high SCD5 expression (top 20%) and low SCD5 expression (bottom 20%). A total of 1923 genes (1315 down-regulated and 608 up-regulated) that had a ∣log2(fold change) ∣ ≥ 1 and a *p* value < 0.05 were considered significantly differentially expressed. Biological annotations and functional enrichment of overlapped DEGs were performed by using Metascape database. The results of GO analysis (Fig. 6) showed that the 608 SCD5-related DEGs (up-regulated in breast cancer with high SCD5 expression) were involved in negative regulation of cell cycle (the most significant biological process). In addition, these DEGs were also involved in cell division, DNA repair and so on. Taken together, SCD5 might serve as a tumor suppression gene and negatively regulate cancer cell growth and proliferation in breast cancer.

In order to further understand the role of SCD5 in cell cycle, we explored correlations between SCD5 expression and several cell cycle regulators using dataset from Oncomine (Hatzis Breast). Uncontrolled cell proliferation is defining the feature of tumor cells. Cell cycle progression is coordinately controlled by many protein factors, which mainly include cyclins, cyclin-dependent kinases (CDKs), cyclin-dependent kinase inhibitors (CKIs), etc. The key to cell cycle regulation is CDK activation during specific cell cycle changes^35^. Most of the known cyclin-dependent kinases (CDKs) could regulate cell cycle progression. There are at least nine isoforms of CDKs in animal cells, among which four isoforms (CDK1-4) are directly involved in the regulation of cell cycle. In mammalian cells, CDK1 interacts with cyclin to drive cell cycle progression. Another isoform, CDK7, is involved in the activation of cyclin dependent kinase activated kinase^36^. Analyses revealed that SCD5 expression was negatively correlated with multiple cell cycle regulators (Table S1).

## Discussion

Currently, NACT has become a major trend in clinical management of breast cancer. Improved response to NACT is related to better survival outcomes. However, there are still no effective biomarkers used to predict the responsiveness and efficacy of neoadjuvant chemotherapy in breast cancer patients, especially TNBC patients. Therefore, it is necessary to identify novel biomarkers to predict the response to NACT. Previous study showed that the SCD5 expression in patients who achieved pCR after neoadjuvant chemotherapy was lower than that in patients who did not achieve pCR^15^. Here, we revealed that SCD5 could be a potential predictor of response to neoadjuvant therapy, especially in TNBC by using public databases.

Dysregulation of lipid metabolism is considered as a component of malignant transformation in many different cancers, including breast cancer^37^. The balance between saturated and monounsaturated fatty acids could regulate cell growth and differentiation through affecting cell membrane fluidity and signal transduction^38^. SCDs was known as the rate-limiting enzyme catalyzing the synthesis of monounsaturated fatty acids^39,40^. Two isoforms of SCDs (SCD1 and SCD5) are characterized in human^9^. It is well recognized that SCD1 expression is up-regulated in cancer cells which could promote the cell proliferation and increase tumor membrane fluidity^10^. Different from the role of SCD1 in human cancers, our data revealed that SCD5 had differential expression profiles in human cancers, and in breast cancer, SCD5 was upregulated in normal tissue compared to tumor. There was no significant correlation between SCD1 and SCD5 expression in different human cancer types. Furthermore, we found that reduced SCD5 expression was correlated with some aggressive features, such as high histological grade, late stage, HER2 overexpression, BRAF, KRAS and CDKN2A mutations, and shorter RFS. Unlike SCD5, we did not observe obvious clinical significance of SCD1 in breast cancer by using public databases. Taken together, the results showed that the two SCDs isoforms might paly separate roles in breast cancer. Analysis of KEGG pathway showed that SCD5 was involved in peroxisome proliferator-activated receptor (PPAR) and AMP-activated protein kinase (AMPK) signaling pathways (Table 2), which were related to the inhibition of the multiplication of tumor cell. Results of GO annotation and functional enrichments showed that SCD5-related DEGs were mainly involved in negative regulation of cell cycle cell division and DNA repair. These findings are similar to observations in melanoma. Bellenghi et al. proved that the restored expression of SCD5 and its production- oleic acid could protect against malignancy in melanoma, especially during the metastatic dissemination phase by driving advanced melanoma cell lines toward differentiation and reversion of epithelial-mesenchymal^12,13^. We further analyzed the correlation between HER2 and SCDs expression. The results showed that SCD5 expression was negatively correlated to HER2 expression, and SCD1 expression was positively correlated to HER2 expression. The former research confirmed that monounsaturated fatty acid could suppress HER2 expression in breast cancer^41^, which might explain the negative correlation between SCD5 and HER2 expression as well as the higher SCD5 expression in TNBC compared with other molecular subtypes. However, it could not explain the reason why SCD1 expression was positively related to HER2 expression, which needs further exploration. Stated thus, these data suggested that SCD5 might serve as a regulator of diverse signaling networks to suppress cell proliferation and migration in breast cancer.

Moreover, our data also suggested that SCD5 could be a potential predictor of response to anthracycline/taxane-based neoadjuvant chemotherapy in patients with breast cancer, especially in TNBC patients. Our data showed that SCD5 expression was negatively correlated with multiple cell cycle regulators. The clinical used cytotoxic drugs such as paclitaxel and vinblastine are cell cycle specific agents (CCSA), which are only sensitive to certain phases of the cell proliferation cycle, but not to G0 phase cells. Thus, we hypothesized that high SCD5 expression might induce cell cycle checkpoint arrest and lead to chemoresistance. In addition, Wang et al. observed that fatty acid oxidation rate was significantly increased in paclitaxel-resistant TNBC (MDA-MB 231) cells and indicated that fatty acids oxidation was required for the maintenance of breast cancer stem cells and contributes to chemoresistance^42^. Because of the absence of double bonds, saturated fatty acids are more stable than unsaturated fatty acids. With the increase of unsaturated double bond, the fatty acids become more unstable and easier to be oxidized^43^. We speculated that SCD5 might catalyze the formation of unsaturated fatty acids from saturated fatty acids, which increase the fatty acid oxidation rate and thus lead to chemoresistance. The mechanism of this part needs further study. In addition, our data observed that SCD5 was significantly involved in AMPK signaling pathway, which was related to cancer drug resistance^34^. Taken together, SCD5 expression signatures could be associated with response of neoadjuvant chemotherapy in breast cancer.

In summary, our study was the first to investigate the roles of SCD5 in breast cancer. The results showed that SCD5 might serve as a tumor suppressor gene during breast cancer progression. Furthermore, SCD5 might help distinguish breast cancer patients, especially TNBC patients, who are likely to benefit from neoadjuvant chemotherapy.

## Supplementary Information

Supplementary Figure 1.

Supplementary Figure 2.

Supplementary Figure 3.

Supplementary Figure 4.

Supplementary Figure 5.

Supplementary Table 1.

Supplementary Information.

## Data Availability

All data is provided in the manuscript.

## References

[1] Sung HG (2020). Global cancer statistics 2020: GLOBOCAN estimates of incidence and mortality worldwide for 36 cancers in 185 countries. CA Cancer J. Clin..

[2] Brown M, Tsodikov A, Bauer KR, Parise CA, Caggiano V (2008). The role of human epidermal growth factor receptor 2 in the survival of women with estrogen and progesterone receptor-negative, invasive breast cancer: the California Cancer Registry, 1999–2004. Cancer.

[3] von Minckwitz G (2012). Definition and impact of pathologic complete response on prognosis after neoadjuvant chemotherapy in various intrinsic breast cancer subtypes. J. Clin. Oncol..

[4] Liedtke C (2008). Response to neoadjuvant therapy and long-term survival in patients with triple-negative breast cancer. J. Clin. Oncol..

[5] Cortazar P (2014). Pathological complete response and long-term clinical benefit in breast cancer: the CTNeoBC pooled analysis. Lancet.

[6] Kong X, Moran MS, Zhang N, Haffty B, Yang Q (2011). Meta-analysis confirms achieving pathological complete response after neoadjuvant chemotherapy predicts favourable prognosis for breast cancer patients. Eur. J. Cancer.

[7] Early Breast Cancer Trialists' Collaborative, G (2018). Long-term outcomes for neoadjuvant versus adjuvant chemotherapy in early breast cancer: meta-analysis of individual patient data from ten randomised trials. Lancet Oncol..

[8] Zaidi N (2013). Lipogenesis and lipolysis: the pathways exploited by the cancer cells to acquire fatty acids. Prog. Lipid Res..

[9] Wang J (2005). Characterization of HSCD5, a novel human stearoyl-CoA desaturase unique to primates. Biochem. Biophys. Res. Commun..

[10] Scaglia N, Igal RA (2005). Stearoyl-CoA desaturase is involved in the control of proliferation, anchorage-independent growth, and survival in human transformed cells. J. Biol. Chem..

[11] Angelucci C (2018). Pivotal role of human stearoyl-CoA desaturases (SCD1 and 5) in breast cancer progression: oleic acid-based effect of SCD1 on cell migration and a novel pro-cell survival role for SCD5. Oncotarget.

[12] Bellenghi M (2015). SCD5-induced oleic acid production reduces melanoma malignancy by intracellular retention of SPARC and cathepsin B. J. Pathol..

[13] Puglisi R (2018). SCD5 restored expression favors differentiation and epithelial-mesenchymal reversion in advanced melanoma. Oncotarget.

[14] Xu Y (2019). Identification of differentially expressed genes and functional annotations associated with metastases of the uveal melanoma. J. Cell. Biochem..

[15] Chen YZ (2012). PPAR signaling pathway may be an important predictor of breast cancer response to neoadjuvant chemotherapy. Cancer Chemother. Pharmacol..

[16] Clough E, Barrett T (2016). The gene expression omnibus database. Methods Mol. Biol..

[17] Deng M, Bragelmann J, Schultze JL, Perner S (2016). Web-TCGA: an online platform for integrated analysis of molecular cancer data sets. BMC Bioinform..

[18] Thul PJ, Lindskog C (2018). The human protein atlas: a spatial map of the human proteome. Protein Sci..

[19] Rhodes DR (2004). ONCOMINE: a cancer microarray database and integrated data-mining platform. Neoplasia.

[20] Hou GX, Liu P, Yang J, Wen S (2017). Mining expression and prognosis of topoisomerase isoforms in non-small-cell lung cancer by using Oncomine and Kaplan–Meier plotter. PLoS ONE.

[21] Kanehisa M (2019). Toward understanding the origin and evolution of cellular organisms. Protein Sci..

[22] Kanehisa M, Goto S (2000). KEGG: kyoto encyclopedia of genes and genomes. Nucleic Acids Res..

[23] Kanehisa M, Furumichi M, Sato Y, Ishiguro-Watanabe M, Tanabe M (2021). KEGG: integrating viruses and cellular organisms. Nucleic Acids Res..

[24] Perou CM (2000). Molecular portraits of human breast tumours. Nature.

[25] Slamon DJ (1987). Human breast cancer: correlation of relapse and survival with amplification of the HER-2/neu oncogene. Science.

[26] Hayes DF (2007). HER2 and response to paclitaxel in node-positive breast cancer. N. Engl. J. Med..

[27] Ueno NT, Yu D, Hung MC (1997). Chemosensitization of HER-2/neu-overexpressing human breast cancer cells to paclitaxel (Taxol) by adenovirus type 5 E1A. Oncogene.

[28] Muss HB (1994). c-erbB-2 expression and response to adjuvant therapy in women with node-positive early breast cancer. N. Engl. J. Med..

[29] Dressler LG (2005). Comparison of HER2 status by fluorescence in situ hybridization and immunohistochemistry to predict benefit from dose escalation of adjuvant doxorubicin-based therapy in node-positive breast cancer patients. J. Clin. Oncol..

[30] Nickkho-Amiry M, McVey R, Holland C (2012). Peroxisome proliferator-activated receptors modulate proliferation and angiogenesis in human endometrial carcinoma. Mol. Cancer Res..

[31] Li W, Saud SM, Young MR, Chen G, Hua B (2015). Targeting AMPK for cancer prevention and treatment. Oncotarget.

[32] Bougarne N (2018). Molecular actions of PPARalpha in lipid metabolism and inflammation. Endocr. Rev..

[33] He L (2017). AMPK regulation of glucose, lipid and protein metabolism: mechanisms and nutritional significance. Curr. Protein Pept. Sci..

[34] Wang Z, Wang N, Liu P, Xie X (2016). AMPK and cancer. Exp. Suppl..

[35] Santo L, Siu KT, Raje N (2015). Targeting cyclin-dependent kinases and cell cycle progression in human cancers. Semin. Oncol..

[36] Satyanarayana A, Kaldis P (2009). Mammalian cell-cycle regulation: several Cdks, numerous cyclins and diverse compensatory mechanisms. Oncogene.

[37] Baumann J, Sevinsky C, Conklin DS (1831). Lipid biology of breast cancer. Biochim. Biophys. Acta.

[38] Ntambi JM, Miyazaki M (2004). Regulation of stearoyl-CoA desaturases and role in metabolism. Prog. Lipid Res..

[39] St John LC, Lunt DK, Smith SB (1991). Fatty acid elongation and desaturation enzyme activities of bovine liver and subcutaneous adipose tissue microsomes. J. Anim. Sci..

[40] Rincon G (2012). Polymorphisms in genes in the SREBP1 signalling pathway and SCD are associated with milk fatty acid composition in Holstein cattle. J. Dairy Res..

[41] Menendez JA, Vellon L, Colomer R, Lupu R (2005). Oleic acid, the main monounsaturated fatty acid of olive oil, suppresses Her-2/neu (erbB-2) expression and synergistically enhances the growth inhibitory effects of trastuzumab (Herceptin) in breast cancer cells with Her-2/neu oncogene amplification. Ann. Oncol..

[42] Wang T (2018). JAK/STAT3-regulated fatty acid beta-oxidation is critical for breast cancer stem cell self-renewal and chemoresistance. Cell Metab..

[43] Leyton J, Drury PJ, Crawford MA (1987). Differential oxidation of saturated and unsaturated fatty acids in vivo in the rat. Br. J. Nutr..

