# Supplementary figures and images for: SCD5 expression correlates with prognosis and response to neoadjuvant chemotherapy in breast cancer
Source: Sci Rep. 2021 Apr 26;11:8976. doi: 10.1038/s41598-021-88258-9 (PMC8076324; doi:10.1038/s41598-021-88258-9)

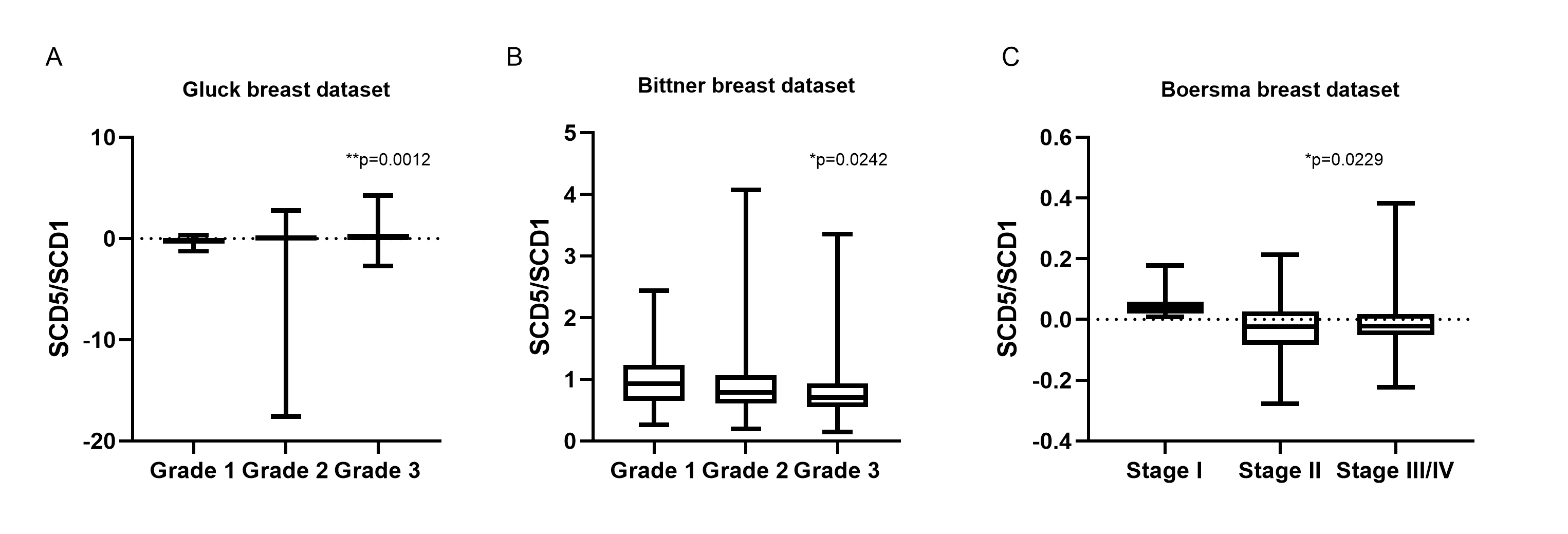

Supplement: Supplementary file 2 — Supplementary Figure 2. [file 41598_2021_88258_MOESM2_ESM.tif]

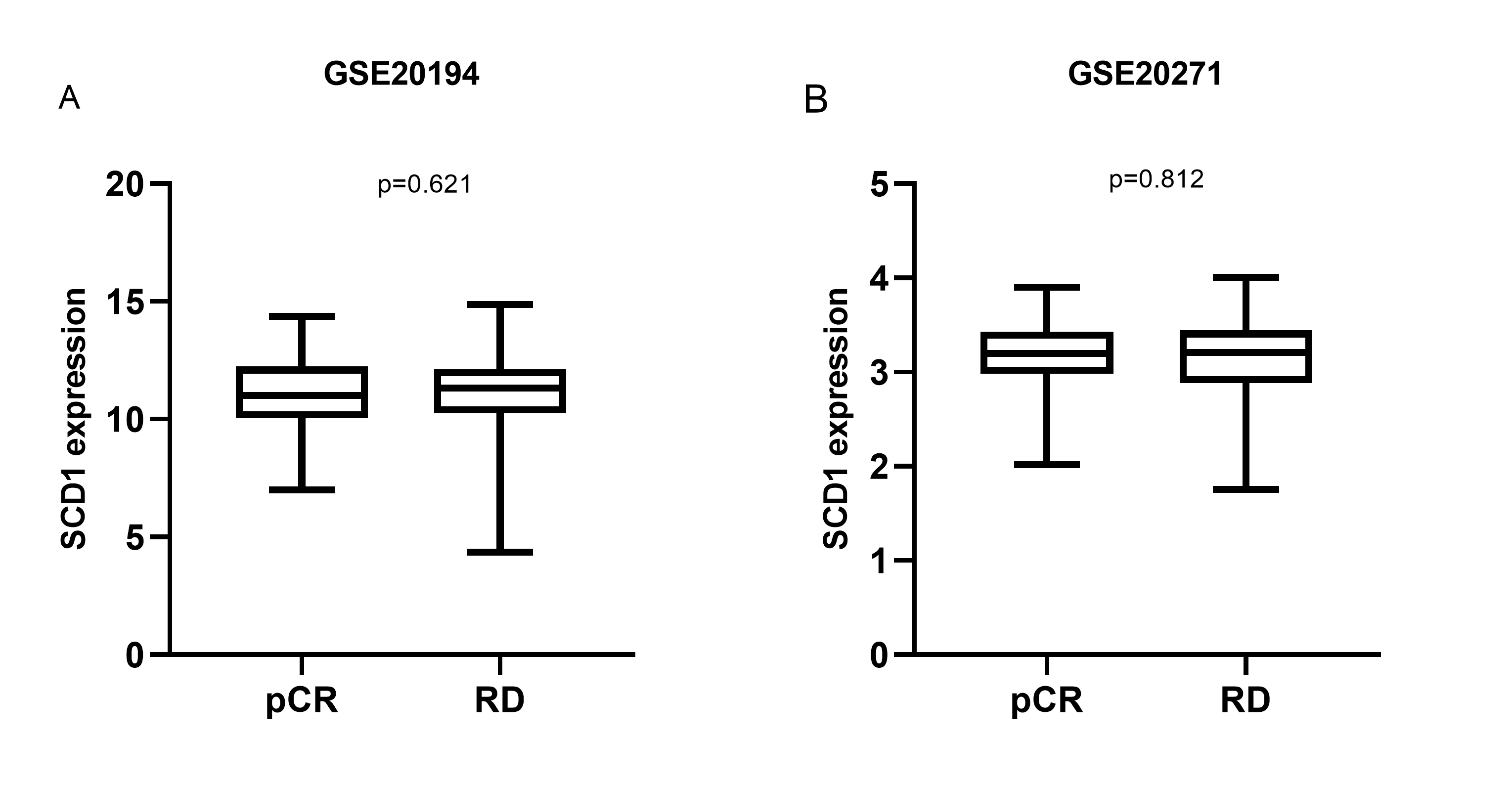

Supplement: Supplementary file 4 — Supplementary Figure 4. [file 41598_2021_88258_MOESM4_ESM.tif]

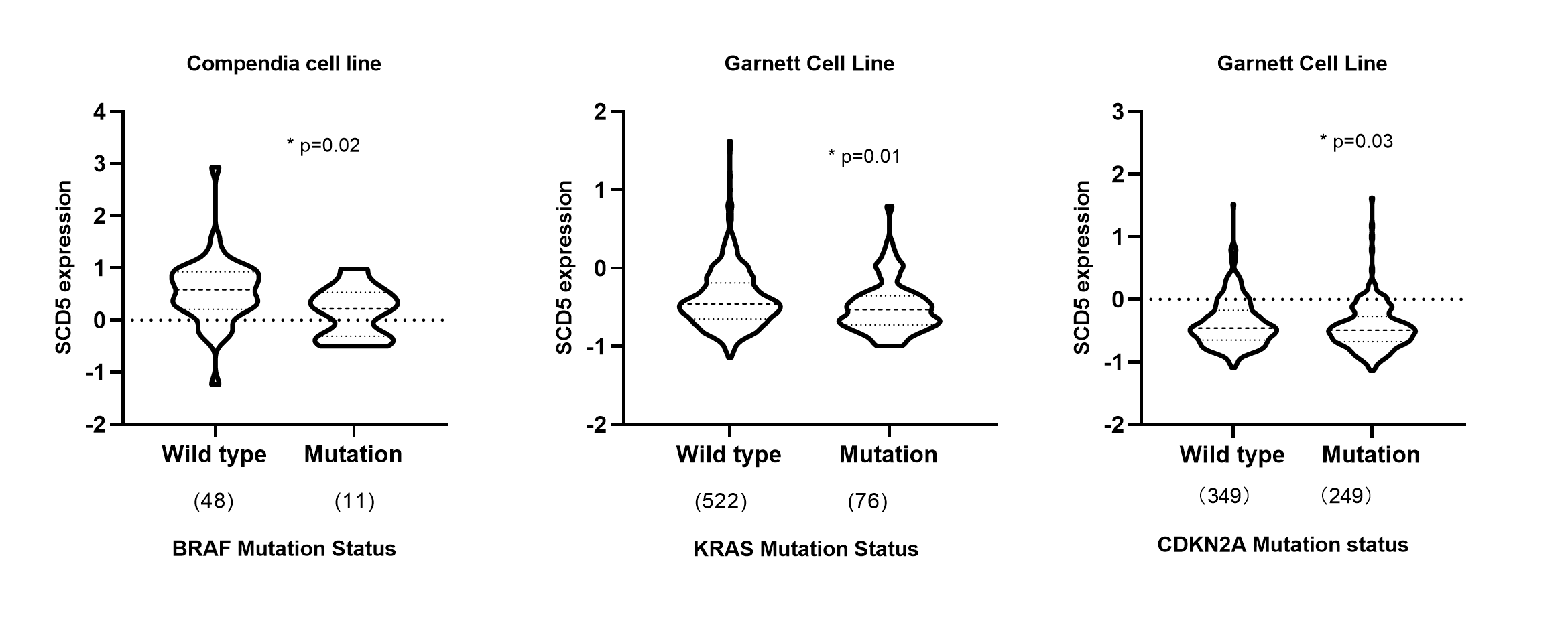

Supplement: Supplementary file 5 — Supplementary Figure 5. [file 41598_2021_88258_MOESM5_ESM.tif]
