# Supplementary material for: SCD5 expression correlates with prognosis and response to neoadjuvant chemotherapy in breast cancer
Source: Sci Rep. 2021 Apr 26;11:8976. doi: 10.1038/s41598-021-88258-9 (PMC8076324; doi:10.1038/s41598-021-88258-9)
Supplement: Supplementary file 6 — Supplementary Table 1. [file 41598_2021_88258_MOESM6_ESM.docx]

Table S1 The correlation between SCD5 expression and cell cycle regulators

| Gene name | Pearson r | 95% confidence interval | P-value |
| --- | --- | --- | --- |
| CDK1 | -0.2541 | -0.3337 to -0.1709 | <0.0001 |
| CDK2 | -0.1245 | -0.2093 to -0.03796 | 0.0049 |
| CDK3 | -0.1400 | -0.2243 to -0.05367 | 0.0016 |
| CDK4 | -0.4126 | -0.4823 to -0.3377 | <0.0001 |
| CDK6 | -0.1206 | -0.2054 to -0.03392 | 0.0065 |
| CDK7 | -0.4983 | -0.5610 to -0.4300 | <0.0001 |
| CDK10 | -0.4217 | -0.4907 to -0.3475 | <0.0001 |
| CCND1 | -0.2369 | -0.3174 to -0.1531 | <0.0001 |
| CCND3 | -0.1510 | -0.2349 to -0.06487 | 0.0006 |
| CCNB1 | -0.3667 | -0.4396 to -0.2889 | <0.0001 |
