# Supplementary material for: SCD5 expression correlates with prognosis and response to neoadjuvant chemotherapy in breast cancer
Source: Sci Rep. 2021 Apr 26;11:8976. doi: 10.1038/s41598-021-88258-9 (PMC8076324; doi:10.1038/s41598-021-88258-9)
Supplement: Supplementary file 7 — Supplementary Information. [file 41598_2021_88258_MOESM7_ESM.docx]

Supplementary materials

Table S1 The correlation between SCD5 expression and cell cycle regulators

Figure S1 The mRNA expression of SCD5 in different human organs by using samples from GTEx.

@Manual{,

title = {R: A Language and Environment for Statistical

Computing},

author = {{R Core Team}},

organization = {R Foundation for Statistical Computing},

address = {Vienna, Austria},

year = R4.0.4&2021

url = {https://www.R-project.org}

}

@Manual{,

title = {ggpubr: 'ggplot2' Based Publication Ready Plots},

author = {Alboukadel Kassambara},

year = {2020},

note = {R package version 0.4.0},

url = {https://CRAN.R-project.org/package=ggpubr},

}

Figure S2 Relationship analysis of the ratio SCD5/SCD1 and different breast cancer phenotypes by using datasets from Oncomine.

Figure S3 The relationship between SCDs (SCD1 and SCD5) mRNA expression and HER2 status/ERBB2 mRNA expression.

(A). The correlation between SCD1 mRNA expression and HER2 status/ERBB2 mRNA expression using samples from Oncomine and GEO. (B). The correlation between SCD5 mRNA expression and HER2 status/ERBB2 mRNA expression using samples from Oncomine and GEO.

Figure S4 Relationship between SCD1 mRNA expression and response to neoadjuvant chemotherapy.

Figure S5 Correlation analysis between mRNA expression of SCD5 and oncogene/ antioncogene mutation. A. SCD5 mRNA expression in BRAF wild type patients and mutation carriers using Compendia cell line from Oncomine. B. SCD5 mRNA expression in KRAS wild type patients and mutation carriers using Garnett cell line from Oncomine. C. SCD5 mRNA expression in CDKN2A wild type patients and mutation carriers using Garnett cell line from Oncomine.
